# The Association of Agent Orange (AO) Exposure with Monoclonal Gammopathy of Undetermined Significance (MGUS) to Multiple Myeloma (MM) Progression: A Population-based Study of Vietnam War Era Veterans

**DOI:** 10.21203/rs.3.rs-3396573/v1

**Published:** 2023-10-09

**Authors:** Lawrence W. Liu, Mei Wang, Nikhil Grandhi, Mark A. Schroeder, Theodore Thomas, Kristin Vargo, Feng Gao, Kristen M. Sanfilippo, Su-Hsin Chang

**Affiliations:** St. Louis Veterans Affairs Medical Center; St. Louis Veterans Affairs Medical Center; St. Louis Veterans Affairs Medical Center; Washington University School of Medicine; St. Louis Veterans Affairs Medical Center; St. Louis Veterans Affairs Medical Center; Washington University School of Medicine; St. Louis Veterans Affairs Medical Center; St. Louis Veterans Affairs Medical Center

## Abstract

**Background:**

Herbicide and pesticide exposure (e.g., agent orange [AO]) is associated with an increased risk of multiple myeloma (MM) due to the contaminant, 2,3,7,8-tetrachlorodibenzo-p-dioxin (TCDD). Monoclonal gammopathy of undetermined significance (MGUS) is the precursor state to MM; however, not all patients with MGUS progress to MM. It is unclear whether AO exposure increases the risk of progression of MGUS to MM.

**Purpose:**

We aimed to determine the association between AO exposure and progression to MM in a nation-wide study of U.S. Veterans with MGUS.

**Patients and Methods:**

This is a population-based cohort study of Vietnam Era Veterans diagnosed with MGUS. A natural language processing (NLP) algorithm was used to confirm MGUS and progression to MM. The association between AO and progression was analyzed using multivariable Fine-Gray subdistribution hazard model with death as a competing event. Veterans who served during the Vietnam War Era from 1/9/1962-5/7/1975 and were diagnosed with MGUS between 10/1/1999-12/31/2021 were included. We excluded patients with missing BMI values, progression within 1 year after MGUS diagnosis date, non-IgG or IgA MGUS, or birth years outside of the range of the AO exposed group, and race other than Black and White. AO exposure and service during 1/9/1962-;5/7/1975 and stratified according to TCDD exposure levels by three time periods: 1/9/1962-11/30/1965 (high), 12/1/1965-12/31/1970 (medium), or 1/1/1971-5/7/1975 (low). The association between AO and progression was analyzed using multivariable Fine-Gray subdistribution hazard model with death as a competing event.

**Results:**

We identified 10,847 Veterans with MGUS, of whom 7,996 had AO exposure. Overall, 7.4% of MGUS patients progressed to MM over a median follow-up of 5.2 years. In multivariable analysis, AO exposure from 1/9/1962–11/30/1965, high TCDD exposure, was associated with an increased risk of progression (adjusted hazard ratio 1.48; 95% confidence interval 1.02–2.16), compared to Veterans with no exposure.

**Conclusions:**

In patients with MGUS, the high Agent Orange exposure time period is associated with a 48% increased risk of progression to multiple myeloma. This suggests that patients with MGUS and prior Agent Orange exposure or occupational exposure to TCDD (eg. Agricultural workers) may require thorough screening for plasma cell dyscrasias.

## Introduction

Monoclonal Gammopathy of Undetermined Significance (MGUS) is a premalignant plasma cell dyscrasia often found during workup of protein gaps, anemia and kidney disease. It is characterized by serum monoclonal protein (M-protein) < 3 g/dL, < 10% monoclonal plasma cells in the bone marrow, and absence of end organ damage as defined by the International Myeloma Working Group.^1–3^ The incidence of MGUS is 0.1% at 50 years increasing 5-fold by age 90 years.^3^

The risk of progression of MGUS to multiple myeloma (MM) is around 1% per year.^4^ MM is the third most common hematologic malignancy which makes it imperative to identify risk factors for progression from MGUS.^1,3,5^ Low-risk MGUS is defined as: M-protein < 1.5 g/dL, IgG subtype, and normal serum free light chain ratio (FLC), and typically has a risk of progression of 5% over 20 years.^1–4^ Conversely, high-risk MGUS is defined as: M-protein ≥ 1.5 g/dL, non-IgG subtype, and abnormal FLC, and is associated with an absolute risk of progression of 60% over 20 years.^1–4^ Current guidelines recommend yearly laboratory and clinic follow up for MGUS deemed to be high risk and every 2–3 years for low risk MGUS.^6^ Risk factors for progression may identify those who need closer monitoring.

Agent Orange (AO) is an herbicide that was used in chemical warfare during the Vietnam War. The term “Agent Orange” has been broadly used to refer to all the herbicidal agents (i.e., Agents Pink, Green, Purple, White, Orange and Blue) used during the Vietnam War Era (1/9/1962–5/7/1975). A contaminant commonly found in AO and the other herbicidal agents, 2,3,7,8-tetrachlorodibenzo-p-dioxin (TCDD), is its most carcinogenic component.^7–10^ All agents had varying levels of TCDD with Agents Pink, Green, and Purple having almost 16 times the amount compared to AO (up to 66ppm versus 13ppm for AO).^11^ Agents Pink, Green, and Purple were largely used from January 1962 to November 1965 whereas Agent Orange was used from December 1965 to December 1970. From January 1971 to April 1975, these herbicides were no longer used but this period was included in the “AO exposure” definition of the Veterans Health Administration (VHA) given the possibility of residual environmental exposure to expand access for service connection to Veterans.^11,12^ This demonstrates that different time periods within the AO exposure period may have different levels of TCDD exposure and analyzing the risk of MGUS progression could inform cancer prevention.

Prior observational studies have identified pesticide and herbicide exposure, especially in the context of farming, as a risk factor for the development of MGUS and MM.^13–17^ In a prospective cohort study, AO exposure was associated with an increased risk of both MGUS and MM.^10^ The same study further found higher risk with increasing serum concentrations of TCDD. Subsequently, in a small single center cohort study (n = 211) at the Detroit VA Medical Center, AO exposure was associated with an 11-fold increased risk of progression of MGUS to MM.^7^ A recent population-based study of the national VA database did not find an association between AO exposure and MGUS progression via International Classification of Diseases (ICD) codes.^18^

This study aims to rigorously assess the association between degree of TCDD/AO exposure and MGUS progression to MM by conducting a nation-wide study of Vietnam War Era Veterans with a diagnosis-confirming methodology.

## Methods

### Data, study population, and design

A retrospective, cohort study using data from the U.S. VHA system was conducted. We identified patients diagnosed with MGUS from 10/1/1999–12/31/2021 via ICD-10 code, 47.2, then confirmed their diagnosis status and dates by an established natural language processing (NLP) algorithm.^19–22^ Patients were considered to have MGUS if they had M-protein detected on serum protein electrophoresis (SPEP) with immunofixation confirmation. This step is crucial as our prior studies utilizing data abstraction demonstrated that, at best, only 80–84% of patients with ICD codes for MGUS were confirmed to have MGUS on manual chart review.^20^

Institutional Review Boards at both Washington University School of Medicine and Veteran Affairs Saint Louis Healthcare System approved the study.

### Analytic MGUS cohort

**Figure 1.**
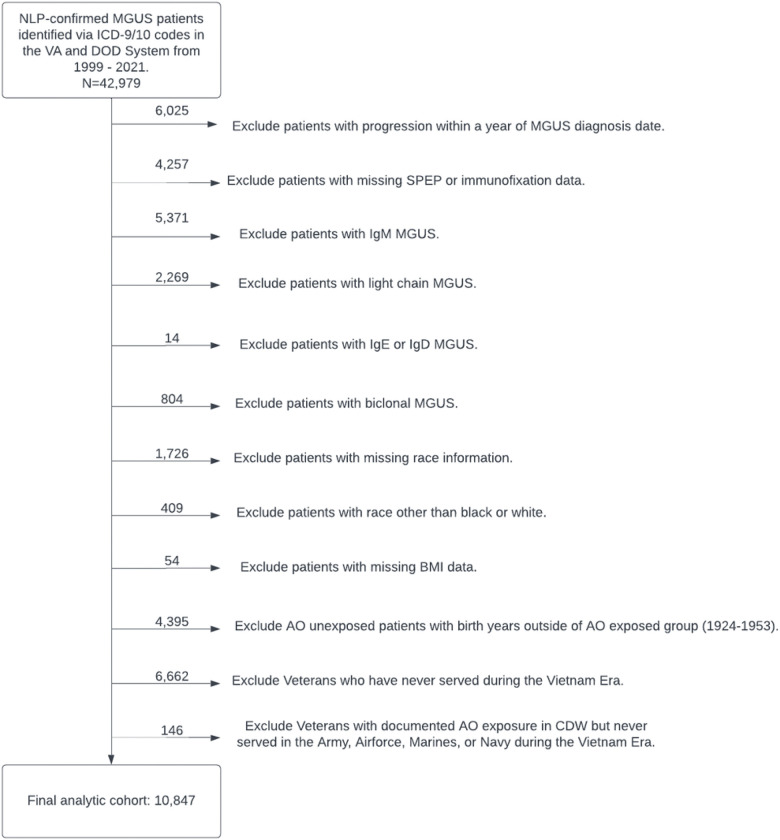
Attrition Diagram.

Veterans with NLP-confirmed MGUS within 1999–2021 who served during the Vietnam War Era between 1/9/1962 and 5/7/1975, the service period acknowledged by VA compensation of benefits for presumption of exposure to AO, were included (Fig. 1).^23^ The date of 1999 was chosen because this was when the Corporate Data Warehouse (CDW) database was launched. Before that year, there was no centralized, electronic storage of diagnostic data so it would not be possible to collect SPEP, immunofixation, and other diagnostic data before that date.^24,25^ We utilized the first entry date and last separation date of the patient service episodes to identify that the Veteran served during the Vietnam War Era. We also excluded Veterans with 1) progression within 1 year after MGUS diagnosis date since patients with progression within 1 year may have had MM at MGUS diagnosis; 2) missing SPEP or immunofixation data; 3) MGUS types other than IgG and IgA because IgM MGUS does not typically progress to MM and light chain MGUS does not reliably show up on SPEP; 4) missing race; 5) race other than black or white due to low sample size; 6) missing BMI data; 7) documented AO exposure but never served in the Army, Airforce, Marines, or Navy during the Vietnam Era; 8) no AO exposure whose birth years were outside of the range of the birth years for the AO exposed group; 8) service time outside of the Vietnam Era (01/09/1962–12/31/1970); and 9). The reason for the last two exclusion criteria was to ensure that the two groups with and without AO exposure are similar in age and timeframe of service. The latter is to ensure that the two groups had comparable follow-up time by restricting AO unexposed patients to have the same service time as the exposed groups.

Unique patient identifiers were used to collect data on type of MGUS, race, sex, M-protein levels, weight, height, and comorbidities at the time of MGUS diagnosis, as well as AO exposure (see below). The most frequently reported height and weight within 1 month of MGUS diagnosis was used for BMI calculation. BMI categories were underweight (BMI < 18.5 kg/m^2^), normal weight (BMI 18.5–24.9 kg/m^2^), overweight (BMI 25–29.9 kg/m^2^), or obese (BMI ≥ 30 kg/m^2^).

### AO exposure

Data on prior AO exposure for the MGUS cohort were obtained from the VHA CDW Patient 3.1 Domain which utilized the station and time period of service to determine exposure.^26^ The Military Service Episode Legacy section was used to identify the branch of service and location of stationing. Duration of exposure was defined as the length of stationing in an area designated as having AO exposure during the Vietnam War Era.^27^ Patients were designated as potentially having AO exposure for this study if they met all of the following criteria: AO exposure documented in CDW, AO location was designated as Vietnam, and if they ever served in the army, air force, navy, or marine corps during the date range of 1/9/1962–5/7/1975.

These patients were further split into three groups depending on their majority time of stationing: 1/9/1962–11/30/1965 (high-exposure), 12/1/1965–12/31/1970 (medium-exposure), and 1/1/1971–5/7/1975 (low-exposure). The reasoning for separating into these three time periods and categorizing the first two as high- and medium- respectively was that the herbicidal agents used in the first time period had the highest levels of TCDD, followed by the second time period.^11,12^ The last time period was when the herbicidal agents were no longer used. For this reason, patients whose entire time of stationing fell in the last time period were categorized as no exposure. Patients whose time of stationing includes the first and/or the second time period but the majority time fell into the third time period were categorized in to the low exposure group, as these Veterans may have had minimal TCDD exposure.^11,12^

### Outcome measures

The outcome was time from MGUS diagnosis to progression to MM. The progression status and dates were detected by identifying patients in the analytic cohort who had an ICD-10 code for MM, C90.0, and also received MM-specific treatment (see Supplemental Table 1). Progression was then further confirmed by a published NLP algorithm^19,20^ by capturing mentioning of MM diagnosis in the clinical notes and bone marrow biopsies (BMBx) with 10% or more plasma cells and/or SPEP demonstrating 3 g/dL or more M-protein.^28^ These confirmation steps were included due to prior studies demonstrating that ICD codes for MM may be only 58% accurate in capturing true MM.^29^ Follow-up was determined by the time of MGUS diagnosis to MM diagnosis, death, or being censored at the time of study (2/7/2023).

### Statistical analyses

Summary statistics of the demographic and clinical characteristics stratified by AO exposure status (high, medium, low, and none) were computed. To compare patients across these four groups, for categorical variables, we used chi-square tests to examine differences in proportions; for continuous variables, we used Student t tests to examine differences in means and Kruskal-Wallis tests to examine the differences in medians. To compare the cumulative incidence of progression between the AO exposure groups, the Gray’s test was performed with death as a competing event.

The association of the AO exposure with progression was estimated using a multivariable Fine-Gray subdistribution hazard model with death as the competing event while adjusting for known covariates associated with progression. These covariates included: service branch, age at MGUS diagnosis, gender, race (White [reference], Black), M-protein (> 1.5, ≤ 1.5 g/dL [reference]), MGUS subtype (Ig A, and Ig G [reference]), BMI category (underweight, normal [reference], overweight, obese), diabetes mellitus (DM), and Charlson comorbidity index (CCI), all at time of MGUS diagnosis.

All tests were two-sided. Statistical significance was determined by an alpha level of 0.05. All statistical analyses were performed using SAS version 9.2 (SAS Institute Inc., Cary, NC).

## Results

After applying our NLP algorithm to confirm MGUS patients identified from ICD-9/10 codes from 1999–2021, we identified 42,979 patients (Fig. 1). We excluded patients with progression within 1 year of MGUS diagnosis date (6,025), missing SPEP data (4,257), non-IgA/IgG MGUS (8,458, which includes IgM [5,371], Light Chain [2,269], IgE [6], IgD [8], or Biclonal [804] subtype), missing race (1,726), race other than Black or White (409), missing BMI data (54), no AO exposure and birth years outside of the birth year range of the AO exposed group (4,395), no service during the Vietnam Era (6,662), and documented AO exposure in CDW but never served in the Army, Airforce, Marines, or Navy during the Vietnam Era (146). This led to our final analytic cohort of 10,847 Veterans.

Of these patients, there were 292 (2.7%) patients in the high AO exposure group, 2,314 (21.3%) patients in the medium AO exposure group, 245 (2.3%) patients in the low AO exposure group, and 7,996 (73.7%) in the group without AO exposure (Table 1). The median duration of exposure was 1,095 days in the high exposure group, 923 days in the medium exposure group, 1,102 days in the low exposure group, and 731 days in the group without AO exposure. The median overall survival (mOS) was 69.2 months in high exposure group, 67.6 months in the medium exposure group, 61.0 months in the low exposure group, and 66.4 months in the unexposed group (P = 0.25).

The four groups were different in progression rate (P < 0.0001), gender (P < 0.0001), race (P < 0.0001), BMI at MGUS diagnosis (P < 0.0001), age at MGUS diagnosis (P < 0.0001), CCI (P = 0.004), AO exposure duration (P < 0.0001), and service branch (P < 0.0001). The median age of MGUS diagnosis was 71.7 years in the high exposure group, 68.6 years in the medium exposure group, 66.9 years in the low exposure group, and 68.7 years in the group without AO exposure (P < 0.0001). The four groups were not statistically significantly different in proportion of patients with M-spike ≥ 1.5 g/dL (P = 0.08), Ig subtype (P = 0.84), follow-up time (P = 0.76), median overall survival (mOS; P = 0.25).

**Figure 2.**
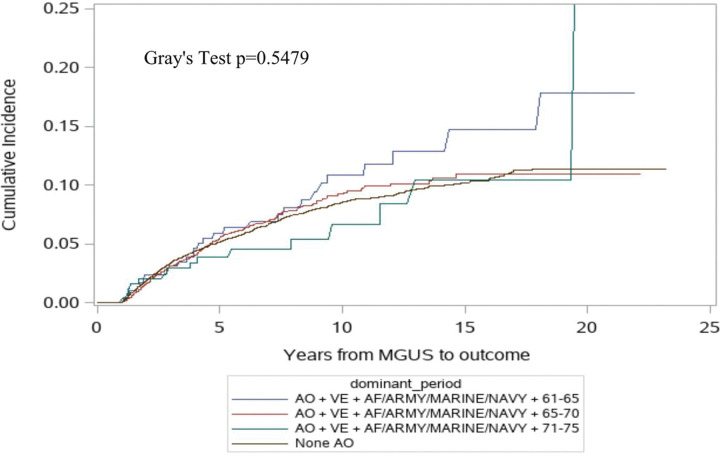
Cumulative Incidence of Progression

Among the analytic MGUS cohort, 9.6% in the high exposure group, 7.3% in the medium exposure group, 6.1% in the low exposure group, and 7.4% in the AO unexposed group progressed (P < 0.0001). However, no statistically significant difference in the cumulative incidence of progression was detected (p = 0.54, Fig. 2). However, in the multivariable analysis, the high exposure group had a significant increase in the risk of progression from MGUS (multivariable-adjusted Hazard Ratio [aHR] 1.48; 95% Confidence Interval [CI] 1.02–2.16; Table 2) compared to those with no AO exposure. There were no significant differences in progression risk noted in the medium (aHR 1.05; 95% CI 0.89–1.25) and the low (aHR 0.83; 95% CI 0.50–1.40) exposure groups compared to no AO exposure.

## Discussion

This study utilized the nationwide VHA database and an NLP-algorithm for diagnosis confirmation to examine the association between AO exposure and MGUS progression. Our findings demonstrate that the period of herbicidal spraying with the highest concentrations of TCDD (1/9/1962–11/30/1965) is associated with an increase in the risk of progression of MGUS to MM. To our knowledge, this is the only nationwide study utilizing a rigorous study design to assess the association between AO exposure and MGUS progression, including diagnosis and progression confirmation. The inclusion and exclusion criteria were designed to allow for fair comparison, and the exposure groups were well-defined based on TCDD concentrations in literature.

A study by Dodlapati et al., recently reported that AO exposure in Veterans was not associated with MGUS to MM progression utilizing ICD-9/10 codes.^18^ However, our study is different from the prior study in a few ways. Given the low accuracy of ICD codes for MM (only 58% of VHA patients with ICD codes for MM are receiving active MM treatment),^29^ we utilized a few critical verification steps. First, all patients with ICD codes for MGUS were scanned by our NLP algorithm to ensure that they have SPEP and immunofixation data supporting the diagnosis. Second, for diagnosing progression to MM, we only evaluated patients with ICD-10 code for MM and the receipt of MM treatment. Third, we utilized a highly accurate NLP algorithm to confirm that clinical notes and diagnostic data support the MM diagnoses.^19–22^ We adjusted for known risk factors for MGUS progression including M-protein level, MGUS subtype, and obesity and for the competing risk of death in the analyses. At present, MGUS is managed by annual to every 2–3 year follow up with SPEP and/or serum free light chains.^1,2^ Our findings support more frequent monitoring of such blood tests in patients with AO exposure given the higher risk of progression.

Previously, MGUS was not a service-connected condition for Veterans who had AO exposure. Based on our results and others demonstrating that AO exposure increases the risk of MGUS progression, MGUS was recently added the list of service-connected disabilities for Veterans who have had AO exposure.^10,30^

AO and other herbicidal agents had varying levels of TCDD, the most carcinogenic component. ^7–10^ Agents Pink, Green, and Purple, used from January 1962 to November 1965, have some of the highest recorded levels.^11,12^ Our results suggest that there may be a dose-dependent relationship between higher levels of TCDD exposure and progression as our study shows that the high exposure group (1/9/1962 and 11/30/1965) has the highest risk of progression. Although AO is no longer used, TCDD is still found in many pesticides, herbicides, and other manufactured chemicals.^11,12^ Prior studies have identified pesticide and herbicide exposure in farmers as risk factors for developing MGUS and MM.^13–17^ Our results suggest that examining the association between TCDD exposure from farming and MGUS progression is urgently warranted. Based on these results, any patients with MGUS and occupational TCDD exposure may warrant more frequent follow up.

The strengths of our study are that we utilized the national VHA database which allowed for a large population ensuring a sufficiently powered study. Patients were not just identified via ICD codes but also confirmed via a validated NLP algorithm that used diagnostic criteria for MGUS and progression for confirmation.^19–22^ This increases the accuracy of outcome measurement compared to the currently published studies. Additionally, AO exposure levels were based on historical documentation of time periods of spraying with various types of herbicidal agents and their TCDD concentrations.^11,12^ This allowed us to study the highest period of AO exposure and identify the association with the highest risk of progression to MM. This highlights the emphasis on prevention that higher level of TCDD exposure is associated with an increased risk of progression and impacts current MGUS patients with occupational exposure to TCDD (agricultural workers).

This study has limitations. Although our study compared the time from MGUS diagnosis to progression, it is likely that the time of MGUS diagnosis is earlier than clinically identified. Moreover, our study could not assess the mechanism of AO exposure on progression to MM. We were unable to assess individual dose-dependence of AO exposure on progression to MM since we did not have access to the details of each Veterans’ work while stationed in Vietnam and serum levels of TCDD were not routinely collected during the Vietnam War. However, a prior study demonstrated the dose dependence of TCDD in the development of MGUS which would support the existence of a similar dose-dependence in progression risk with AO exposure.^10^ While AO is no longer widely used as an herbicide, the carcinogenic component, TCDD, is still an agent that agricultural workers encounter and thus gives modern implications to our study.^11–15^ Additionally, the VHA patient population is different from the US general population,^31–33^ which would limit the generalizability of our results.

## Conclusions

Our findings demonstrate that Agent Orange exposure between 1/9/1962–11/30/1965 is associated with a 48% increase in the risk of progression of MGUS to multiple myeloma. These findings have significant implications for both Veteran MGUS patients as well as non-Veteran MGUS patients with occupational TCDD exposure and/or identifiable serum TCDD levels since TCDD is still found in herbicides used by agricultural workers.^13–17^ Patients with MGUS and TCDD exposure should be considered for increased frequency of monitoring for progression. Future studies should confirm whether there is a dose-dependence of serum TCDD levels and MGUS progression in patients with Agent Orange or other herbicide exposure.

## Figures and Tables

**Table 1:** Baseline Characteristics of the analytic cohort of MGUS patients stratified by AO exposure status

	Exposure Status^†^					
	High	Medium	Low	Unexposed	Total	P-value^‡^
N	292	2,314	245	7,996	10,847	
%	2.7%	21.3%	2.3%	73.7%	100.0%	
Gender (%)						<.0001
F	0	0.04	0	2.3	1.7	
M	100	99.96	100	97.7	98.3	
Race (%)						<.0001
Black	33.6	30.3	26.5	38.7	36.5	
White	66.4	69.7	73.5	61.3	63.5	
BMI at MGUS (%)						<.0001
Underweight	1.0	1.0	0	1.7	1.5	
Normal weight	19.5	16.9	19.6	21.4	20.4	
Overweight	34.9	36.4	31.0	33.7	34.3	
Obese	44.5	45.6	49.4	43.2	43.9	
M-spike (%)						0.088
<1.5	73.6	74.6	74.7	71.8	72.5	
>=1.5	4.1	5.1	4.9	6.3	6.0	
Missing	21.7	19.3	20.6	21.9	21.5	
Ig subtype (%)						0.844
A	14.7	13.7	12.2	14.0	13.9	
G	85.3	86.3	87.8	86.0	86.1	
Age at MGUS Diagnosis						
Median	71.7	68.6	66.9	68.7	68.7	<.0001
Mean	71.2	68.0	66.0	68.6	68.5	
Std	5.4	5.3	5.1	6.5	6.3	
Comorbidity						0.004
Median	1.0	0.0	0.0	0.0	0.0	
Mean	2.4	1.8	2.0	2.0	2.0	
Std	3.0	2.6	2.9	2.8	2.8	
Ever served in Army during Vietnam Era (%)						<.0001
No	52.1	33.4	39.6	44.5	42.2	52.1
Yes	48.0	66.6	60.4	55.5	57.8	48.0
Ever served in Marine Corps during Vietnam Era (%)						<.0001
No	81.5	84.8	84.1	90.2	88.6	
Yes	18.5	15.2	15.9	9.9	11.4	
Ever served in Air Force during Vietnam Era (%)						<.0001
No	86.0	91.4	85.3	82.8	84.8	
Yes	14.0	8.6	14.7	17.3	15.3	
Ever served in Navy during Vietnam Era (%)						<.0001
No	79.8	90.0	88.6	83.0	84.5	
Yes	20.2	10.0	11.4	17.0	15.5	
Outcome (%)						<.0001
Progression	9.6	7.3	6.1	7.4	7.4	
Death without progression	41.1	31.2	35.5	41.9	39.4	
Censored	49.3	61.5	58.4	50.7	53.1	

† High exposure was during 1/9/1962–11/30/1965, medium exposure was during 12/1/1965– 12/31/1970, and low exposure was during 1/1/1971–5/7/1975.

‡ Chi-square tests were conducted to compare percentages for categorical variables, t-tests were used to compare means for continuous variables, and Kruskal-Wallis test was used to compare medians. Abbreviations: IG, immunoglobulin; BMI, body mass index; M-spike, monoclonal spike; CCI, Charlson Comorbidity Index; mOS, median overall survival.

**Table 2:** Multivariable analysis of MGUS progression.

Parameter	aHR		95% Confidence Interval	P-value
**No Exposure**		(reference)		
**High Exposure**		1.484	1.02-2.16	0.04
**Medium Exposure**		1.054	0.89-1.25	0.55
**Low Exposure**		0.833	0.50-1.40	0.49
**MGUS Age**		0.97	0.96-0.98	<0.0001
**Gender**				
	Male	0.84	0.51-1.37	0.49
**Race**				
	Black	1.23	1.07-1.42	0.004
**BMI Category**				
	Obese	1.29	1.06-1.57	0.012
	Overweight	1.24	1.01-1.51	0.041
	Normal weight	(reference)		
	Underweight	0.58	0.26-1.31	0.19
**M-spike**				
	≥1.5 g/dL	4.37	3.62-5.26	<0.0001
**MGUS Type**				
	IgA	1.53	1.28-1.83	<0.0001
	IgG	(reference)		
**CCI**		0.96	0.93-0.98	0.003

Abbreviations: IG, immunoglobulin; BMI, body mass index; M-spike, monoclonal spike; mOS, median overall survival

## Data Availability

The data came from the Veteran’s Health Administration and is not publicly available.
